# A42 LIVE DONOR LIVER TRANSPLANTATION IN PRIMARY SCLEROSING CHOLANGITIS: AN INDICATOR OF AN ORGAN ALLOCATION SYSTEM NOT ADDRESSING PATIENT NEED

**DOI:** 10.1093/jcag/gwac036.042

**Published:** 2023-03-07

**Authors:** K Zheng, F Onofrio, C Xu, S Chen, W Xu, M Vyas, K Bingham, K Patel, L Lilly, N Selzner, E Jaeckel, C Tsien, A Gulamhusein, G M Hirschfield, M Bhat

**Affiliations:** 1 Faculty of Medicine, University of Toronto; 2 Ajmera Transplant Program, University Health Network,; 3 Ajmera Transplant Program, University Health Network; 4 Biostatistics Department, Princess Margaret Cancer Center; 5 PSC Partners Canada; 6 Division of Gastroenterology and Hepatology, University of Toronto, Toronto, Canada

## Abstract

**Background:**

Liver transplantation is frequently lifesaving for people living with primary sclerosing cholangitis (PSC). However, patients are waitlisted for liver transplant (LT) according to the MELD-Na score, which may not accurately reflect the burden of living with PSC.

**Purpose:**

We sought to describe and analyze the clinical trajectory for patients with PSC referred for LT, in a mixed deceased donor/live donor transplant programme.

**Method:**

This was a retrospective cohort study from November 2012 to December 2019 including all patients with PSC referred for assessment at the University Health Network Liver Transplant Clinic. Patients who required multiorgan transplant or re-transplantation were excluded. Liver symptoms, hepatobiliary malignancy, MELD-Na progression, and death were abstracted from chart review. Competing Risk analysis was used for timing of LT, transplant type, and death.

**Result(s):**

Of 172 PSC patients assessed, 144 (84%) were listed, of whom 106/144 (74%) were transplanted. Mean age was 47.6 years and 66% were male. During follow-up through to 2021, 23/144 (16%) were removed from the waitlist due to infection, clinical deterioration, liver-related mortality or new cancer; 3 had clinical improvement. At the time of listing, 118/144 (81.95%) had a potential Living Donor (pLD) of whom 94 were transplanted: 64 live donor and 30 deceased donor. Patients with pLD had 79% lower mortality (p<0.001), and higher rates of transplantation (80% vs 46%). Exception points were granted to 13/172 (7.5%) patients.

**Conclusion(s):**

In a high-volume North American liver transplant centre, most patients with PSC assessed for transplant were listed and subsequently transplanted. However, this was a consequence of patients engaging in live donor transplantation. Our findings support the concern from patients with PSC that MELD-Na allocation does not adequately address their needs.

**Please acknowledge all funding agencies by checking the applicable boxes below:**

CIHR, Other

**Please indicate your source of funding;:**

This study was supported by PSC Partners Canada, Canadian Institutes of Health Research (CIHR), Toronto General and Western Hospital Foundation.

**Disclosure of Interest:**

None Declared

